# New Methods for Assessing Rapid Changes in Suicide Risk

**DOI:** 10.3389/fpsyt.2021.598434

**Published:** 2021-01-26

**Authors:** Elizabeth D. Ballard, Jessica R. Gilbert, Christina Wusinich, Carlos A. Zarate

**Affiliations:** Section on the Neurobiology and Treatment of Mood Disorders, Intramural Research Program, National Institute of Mental Health, National Institutes of Health, Bethesda, MD, United States

**Keywords:** suicide, neuroimaging, clinical trial, rapid-acting, neurocognitive, ecological momentary assessment

## Abstract

Rapid-acting interventions for the suicide crisis have the potential to transform treatment. In addition, recent innovations in suicide research methods may similarly expand our understanding of the psychological and neurobiological correlates of suicidal thoughts and behaviors. This review discusses the limitations and challenges associated with current methods of suicide risk assessment and presents new techniques currently being developed to measure rapid changes in suicidal thoughts and behavior. These novel assessment strategies include ecological momentary assessment, digital phenotyping, cognitive and implicit bias metrics, and neuroimaging paradigms and analysis methodologies to identify neural circuits associated with suicide risk. This review is intended to both describe the current state of our ability to assess rapid changes in suicide risk as well as to explore future directions for clinical, neurobiological, and computational markers research in suicide-focused clinical trials.

## Introduction

The suicide crisis is a psychiatric emergency, and roughly 900,000 Americans present to the emergency department each year for suicidal thoughts and behaviors (1). In this context, a clear need exists for rapid interventions that reduce suicidal thoughts and risk for suicidal behavior, in particular because the week after psychiatric admission and discharge are the highest risk time for suicidal attempt and death (2). However, few evidence-based treatments exist that are specifically targeted toward an active suicidal crisis, and most psychotherapies and pharmacological treatments for suicide risk have a lag of onset of weeks to months or even years. This gap underscores clinicians' need for evidence-based treatments with a rapid onset of action (hours to days) that could reduce suicide risk in the short term even as they connect their patients to longer-term therapies and resources. Indeed, the discrepancy between the need for immediate clinical treatment and the delayed therapeutic efficacy of available treatments may be partially driving the increasing suicide rate (3), a troubling statistic that may be further impacted by the mental health effects of the COVID-19 pandemic (4).

The existing armamentarium of traditional suicide treatments includes lithium (5), clozapine (6), electroconvulsive therapy (ECT) (7), cognitive therapies (8, 9), and dialectical behavioral therapy (10). However, “next-generation” suicide treatments have emerged that seek to reduce suicide risk over the course of hours to days. One example is ketamine, a glutamatergic modulator that has been associated with rapid reductions in suicidal thoughts within hours (11). Another new treatment approach is crisis response or safety planning, an outgrowth of cognitive behavioral therapy for suicide prevention that is associated with rapid changes in suicidal thoughts within hours (12) and decreased risk of suicide attempt within months (13, 14). Other treatments with potentially rapid effects on suicidal thoughts include transcranial magnetic stimulation (15), magnetic seizure therapy (16), and chronotherapies (17).

While these interventions have the potential to transform the clinical treatment landscape for suicidal individuals, there is also a tremendous research opportunity associated with their use. Specifically, the need to measure the efficacy of rapid interventions has the potential to spur researchers to develop new tools and techniques for use in next-generation clinical trials, with particular emphasis on assessing moment-to-moment changes in suicidal ideation/behaviors using novel digital, neuroimaging, and computational methodologies. In this context, clinical trials of rapid-acting interventions permit the incorporation of biomarkers throughout the study design in an experimental therapeutics approach. For example, innovative assessments, tasks, and neuroimaging methods can be measured both at baseline and after intervention/placebo to examine potential biomarkers of response. Such an approach, which has already been implemented in rapid-acting clinical trials for depression (18), has the potential to further our understanding of both mechanisms of action for suicide-focused treatments as well as elucidate the neural correlates of suicide risk (19).

This review will discuss new directions in the assessment of rapid changes in suicide ideation (SI) and suicide risk. The review begins by discussing concerns related to the current way that rapid changes in SI are clinically assessed. Three emerging areas within the suicide risk literature will then be described that could be used to measure efficacy in future clinical trials of rapid-acting antisuicidal agents. These include: (1) ecological momentary assessment (EMA) and digital phenotyping approaches for identifying real-time markers of change in SI and risk factors; (2) cognitive markers of suicide risk, including implicit biases and markers of decision-making associated with suicide risk that could be used to support clinical trials of rapid-acting interventions; and (3) neurobiological markers of suicidal thoughts and risk that could be used to highlight potential avenues connecting SI to changes in specific neural circuitry.

## Limitations of Current Assessments of Rapid Changes

The literature regarding the assessment of rapid-acting interventions for suicide risk is limited by several factors. These include use of self-report measures to determine suicide risk and shortcomings associated with clinical risk assessments that are not designed to assess rapid changes in SI.

First, suicide assessment instruments predominately rely on the participant's report of SI and suicidal urges. This type of assessment of SI and behaviors introduces extra obstacles that may obscure a complete understanding of the current level of suicide risk. For example, individuals may minimize suicidal thoughts due to concerns about stigma, treatment by healthcare professionals, or hospitalization (20). Those familiar with the psychiatric system may know that if they report suicidal thoughts, they will often receive additional extended psychiatric interviews, check-ins, and potentially more intensive treatment. In fact, most individuals who go on to die by suicide deny SI at their last clinical appointment (21). In addition, the active suicidal state—the point at which an individual is most at risk for suicide attempt—is very short; in one study, most individuals reported that the length of time between deciding to act on suicidal thoughts and beginning a suicide attempt was 30 min or less (22). Therefore, in-the-moment assessment of SI during a clinic visit may not accurately capture suicide risk in the weeks, days, or even hours after assessment.

Second, in addition to relying on self-report measures, few, if any, of the suicide assessment instruments were developed to repeatedly assess rapid changes in suicidal symptoms over short periods of time. For example, most of the literature exploring the anti-suicidal effects of ketamine used single items from the Montgomery-Åsberg Depression Rating Scale (MADRS; specifically, Item 10) (23) and the Hamilton Depression Rating Scale (HAM-D; specifically, Item 3) (24) as well as longer, suicide-specific measures such as the Scale for Suicide Ideation (SSI) (25) and the Columbia Suicide Severity Rating Scale (C-SSRS) (26). While each of these instruments is well-validated for assessing overall suicide risk, none were developed to assess short-term changes in SI. In one review of several ketamine clinical trials, Ballard and colleagues evaluated the MADRS, Beck Depression Inventory (BDI) (27), and HAM-D suicide items as well as the short (five-item) and long version of the SSI in the same participants (28). While there was overall agreement between measures, a particular discrepancy was observed between the five-item SSI (screening items administered to every participant) and the longer version (five screening items plus 17 additional items only administered if the participant reached a particular score) over repeated assessments. Such discrepancies call attention to the need for methodologies capable of addressing these potentially clinically meaningful fluctuations over time.

As a result of these limitations, it has been difficult to link suicidal thoughts and behaviors with specific pathophysiologies. While candidate endophenotypes have been suggested (29), to date, few biological targets have been associated with the active suicide crisis or immediate suicide risk, which presents an impediment to developing new treatments and enabling precision psychiatry. However, this gap also underscores a key opportunity to present new perspectives and develop new methodologies for assessing suicide risk.

## EMA and Digital Phenotyping

Over the last decade, research using EMA on mobile devices has become more prevalent as a method of assessing rapid changes in suicide risk. Unlike the more traditional clinical rating scales discussed above, EMA allows rapid changes in suicide risk to be measured through real-time monitoring of changes in SI and behaviors over time.

In EMA, specific items are queried several times a day following a scheduled signal from the device or around moments of distress or specific thoughts and behaviors. Items can include symptoms such as depressed mood, anxiety, suicidal thoughts, as well as physical or social factors such as sleep, physical activity, or social interactions (30, 31). Because assessments are repeated, EMA data collection has the potential to capture transient instances of SI that may not be reported via more static and less frequent traditional measures. For example, in a sample of adults diagnosed with major depressive disorder (MDD), more than half of the sample reported SI through EMA but did not report it on the SSI asking about that same time period, highlighting a critical weakness associated with retrospective clinical measures (32).

In addition, EMA research can address the interplay between suicide risk factors as well as how symptoms fluctuate over the course of days or even hours. In one EMA study of adults with recent suicidal behaviors, suicide risk factors such as hopelessness, burdensomeness, and loneliness were found to vary significantly throughout the day; however, these risk factors did not predict changes in SI within a time span of 4 to 8 h (33). In contrast, another study that used actigraphy, sleep diaries, and EMA found that shorter sleep and poor sleep quality predicted greater SI on the following day, although the reverse was not found (34). Studies such as these demonstrate that EMA can more precisely measure SI over the course of a day and, when associated with physiological measures, capture a wider range of suicide risk factors.

Given the amount of data collected across many timepoints in EMA datasets, researchers have been using new analytic techniques to better characterize suicide risk. For example, Rath and colleagues examined EMA data using network analysis to understand the relationships between psychological risk factors and SI within short blocks of time (30–120 min) by administering 10 EMAs per day (35). Another study used recurrent neural network modeling to predict SI with longitudinal electronic medical record data and EMA and found that adding EMA improved prediction rates compared with a model that used medical record data alone (36). Potentially relevant machine learning methods have also been employed in suicide risk studies that used electronic medical record data; such methods could make it possible to include hundreds of variables to predict suicide risk and to track changes in predictors over longer periods (37, 38).

Another relatively new way of making sense of large EMA datasets and other real-time monitoring data is digital phenotyping, which is the identification of patterns in data drawn from a variety of sources, including mobile devices, GPS, speech samples, internet and social media activity, keyboard activity, and EMA (39–41). In a study designed to identify phenotypes of suicidal thinking and test the relationships of those phenotypes with recent attempts, Kleiman and colleagues used a latent profile analysis approach with a month's worth of EMA data from adults with a history of recent suicide attempts or SI (41). The investigators found that those with more severe and persistent suicidal thoughts were more likely to have recently attempted suicide. EMA research has also turned toward metadata to predict suicide risk, such as response latency for assessment questions (42). In another study, Mikus and colleagues used recurrent neural networks to model short-term mood changes using adherence and usage data from their EMA datasets—a strategy that combines the use of metadata and machine learning analysis and that could be applied to future suicide research (43).

Beyond EMA, finding patterns in passively collected data has implications for detecting changes in suicide risk outside of participants' awareness, such as detection of smartphone movement, location, ambient light, or text messages (44). One study used natural language processing and deep learning to estimate suicide risk based on patterns in social media activity, but the authors noted that their goal was to find patterns indicating risk over a longer period instead of short-term state changes (45). Much research is still needed in this area, and many ethical concerns surround the use of passively collected data, including privacy, inappropriate clinical use (e.g., for coercive interventions or use without an appropriate evidence base), data security, and forensic use (46). However, with careful navigation of these concerns, such phenotypes could have significant clinical utility and provide a valuable step toward precision psychiatry.

It should be noted that while ample evidence exists that EMA and digital phenotyping are feasible for studying suicidal thoughts and behaviors, there are also important limitations to consider. As with other types of measures, researchers must consider the role of self-report and insight in EMA data. When distress is high, participants may not be as likely to complete their EMAs, which may be critical to understanding risk in the context of rapid changes (33). Similarly, if no suicide attempts occur in the sample during the study period, predictions related to suicide risk must rely on proxy measures, such as existing clinical scales. Furthermore, although these new methods are low-burden for clinicians compared to comprehensive clinical interviews, they are time intensive and repetitive for participants. Compliance across EMA studies is also a longstanding concern, particularly when studies last longer than a few weeks. Recent EMA studies in samples of individuals with SI found compliance rates of around 65% per assessment, although the role of incentives (such as increased compensation for compliance), the length of the study, and the number of daily assessments may affect those rates (33, 47). In addition, concerns about clinical follow-up exist for suicide assessment in outpatient trials; will a clinician respond to each endorsement of clinically significant SI day or night? Such studies will therefore need to have clear boundaries and expectations in the informed consent process about clinical monitoring of responses and when the participant should seek emergency care. Finally, Kleiman and colleagues raised concerns about data overfitting in EMA studies (48); in this context, previously-used machine learning models in suicide research may not be appropriate for real-time monitoring data (38, 49).

Despite these limitations, EMA and real-time monitoring methods make it possible to study rapid changes in suicidal thoughts and behaviors, suicide risk, and associated symptoms without many of the limitations associated with traditional clinical measures. Digital phenotyping and machine learning approaches to this real-time monitoring data could be particularly useful in trials of rapid-acting interventions, both in identifying the efficacy of interventions and predicting who is likely to benefit. Future directions include the development of analytic techniques to interpret rapid changes in this complex data over the course of treatment. Although results from EMA and digital phenotyping approaches are unlikely to be as readily interpretable as results from clinical rating scales (for example, is a patient “suicidal” if they report SI on only one of the 35 assessments they completed during the week?) they may nevertheless reveal latent suicide risk factors such as variability of SI or time spent in the suicidal state, which could serve as valuable metrics of treatment response in future clinical trials.

## Cognitive and Implicit Biases

Behavioral tasks are another way to assess suicide risk beyond self-report. Cognitive constructs such as impulsiveness and hopelessness have already been linked to suicide risk, primarily using self-report measures (50). Behavioral tasks can assess those constructs in real time without the biases of self-report measures. The bulk of the initial literature focused on traditional cognitive measures, including the Iowa Gambling Task, the Stroop, the Go/No-Go, or the Wisconsin Card Sorting Task; results indicated that, on average, suicide attempters exhibit less cognitive flexibility, more impulsivity, and worse memory and attention than non-attempters (50–52). While the extensive literature on cognitive deficits implicated in suicide risk is outside the scope of this review, there is an emerging literature on tasks that might be most relevant for inclusion into a suicide-focused clinical trial. These tasks are suicide-specific, in that they assess potential implicit biases for suicide-related content without relying on self-report measures. Additionally, because of their brevity, they can be easily incorporated into a clinical trial without extensive equipment or training, as required by techniques such as neuroimaging.

The most prominent examples of suicide-specific tasks are formulations of the Implicit Association Task (IAT). Although the IAT was initially developed in the social psychology literature, Nock and colleagues adapted it to measure both non-suicidal self-injury and associations between the self and death/life (53, 54). In the computerized version of the death/life IAT, the participant is asked to categorize a series of words into “death” or “life,” “me” or “not me,” and their respective combinations (i.e., “life or me,” “death or me,” “life or not me,” “death or not me”). Participants who categorize words into the “death or me” categories more quickly than into the “life or not me” categories are considered to have an implicit association with death. This implicit association with death has been found to predict lifetime and future risk of suicide attempt (54, 55) as well as SI over the course of treatment (56). Critically, several versions of the IAT have also been incorporated into clinical ketamine trials, which found that the “escape” version of the IAT (instead of “death,” the participant links the self with “escape” or “stay”) was associated with reductions in SI (57, 58). In addition, changes in reaction time on the suicide-related IAT were found after a mood induction paradigm in suicide ideators but not in non-ideators (59), suggesting that IAT responses are reactive to mood.

Another area of study focuses on attentional biases to suicide-related stimuli, most easily represented by a formulation of the Stroop task with suicide-related stimuli. Specifically, in a “Suicide Stroop,” individuals are asked to name the color of suicide-related negative- or neutrally-valenced words. Initial results demonstrated that increased interference for suicide-related words—indicating a suicide attentional bias—was associated with increased risk for suicide attempt at 6-month follow-up as well as with recency of suicide attempt (60). However, attempts to replicate these findings have been less consistent than results for the suicide-related IAT (61, 62), and treatment effects and reactivity to mood have not been demonstrated (59). Further investigation of attentional biases for suicide—perhaps using other models of attentional bias, such as a dot-probe task or eye tracking—might determine whether such tasks could be useful in the context of suicide-specific clinical trials.

In summary, there is one suicide-specific task, the suicide IAT, which demonstrates initial utility for clinical trials due to its ability to predict later suicidal thoughts and behaviors, while also exhibiting sensitivity to treatment effects. Benefits of these cognitive or task-related approaches are that such tasks are usually relatively quick to administer, low-cost, easily scalable, and do not rely on direct report of suicide-related symptoms. Limitations include the complex nature of many of the tasks, which preclude associating task performance with specific neural circuits. As a result, new computational approaches that model specific task-performance features or components may be informative for assessing suicide risk. For example, in one recent study of the Iowa Gambling Task, Bayesian analysis of decision-making performance demonstrated that suicide attempters exhibited greater loss aversion than depressed controls or healthy volunteers (63); similar approaches to the suicide-specific tasks may provide further insight into how suicide-related biases change after an intervention. Evaluating responses to these behavioral tasks during neuroimaging scans may be another pertinent channel for understanding the neural circuitry of implicit suicidal thoughts or suicide bias, as described in the next section.

## Neurobiological Markers of Suicidal Thoughts and Suicide Risk

Several neuroimaging studies have attempted to characterize SI from a neurobiological perspective. However, no studies to date have assessed rapid, hour-to-hour changes in SI, a significant limitation given the moment-to-moment variability of suicide risk reviewed above. Many studies that have assessed functional brain changes associated with suicide risk have characterized suicide risk using lifetime history of suicide attempt regardless of time since the most recent attempt. In addition, neuroimaging studies have typically characterized the neurobiology of SI or some metric of suicide risk using resting-state scans or tasks developed to assess correlates of mood disorders (e.g., evaluation of emotional faces or decision-making) rather than tasks developed to assess suicide risk directly. Finally, these studies have mostly assessed suicide risk in larger clinical samples of participants diagnosed with mood disorders (64–75) and other psychiatric conditions (76–79) with which suicide is a comorbid phenotype, but rarely have they investigated suicide transdiagnostically. Given these limitations, an urgent need exists to understand the neurobiology associated with rapid changes in suicidal thoughts to better characterize SI trajectories independent of comorbid psychiatric conditions or diagnosis. Here, we focus on the current “state of the art” in terms of what is known about the neurobiology underpinning suicide risk drawn from functional brain imaging studies.

A recent comprehensive review of structural and functional brain changes accompanying suicidal thoughts and behaviors implicated the ventral and dorsal prefrontal cortex systems and their connections with insula, anterior cingulate, mesial temporal structures, striatal, and posterior connection sites with suicide risk (80). Subsequent resting-state studies assessing changes in large-scale functional brain networks associated with suicide risk in adults have reported similar networks of brain regions. For example, resting-state studies of SI in adults diagnosed with MDD have found network changes in the hippocampus, thalamus, caudate (66), and anterior insula (72) associated with SI scores. In addition, altered seed-based connectivity has been found in the hippocampus, thalamus, and temporal lobe (69) as well as the inferior frontal, orbital, and parietal lobes (75). A resting-state study of large-scale brain networks in suicide attempters compared to non-attempters found that increased posterior default mode network connectivity was associated with recent suicidal activity in attempters; attempters also had greater midline circuitry activity and differential basal ganglia and default mode network co-activity (73). Another study of recent attempters found that suicidal behavior was associated with distinct patterns of connectivity within the executive control network and between the executive control, salience, and default mode networks (68). Similar findings of distinct, between-network associations between cross-frequency amplitude-amplitude connectivity in low-frequency (delta and theta) bands and SI have been reported, with particular hubs emerging from the hippocampus, anterior insula, and bilateral dorsolateral prefrontal cortex (81). Furthermore, among young adults diagnosed with MDD, altered connectivity was reported within subregions of the anterior cingulate cortex in suicide attempters compared to non-attempters (74). In a broader psychiatric sample, machine learning applied to resting-state data demonstrated altered network-level connectivity in frontal and temporal regions as well as in the amygdala, parahippocampus, and putamen (77).

Studies in adolescents during rest have also found network-level changes associated with SI in overlapping brain regions. For example, decreased connectivity between the default mode, salience, and executive control networks was reported in adolescents who were currently experiencing depressive symptoms and had a history of suicide attempt, in addition to increased connectivity between the salience and executive control networks (71). Longitudinal decreases in SI have also been associated with increased salience network coherence in depressed adolescents (82). Moreover, a study of adolescents with internet addiction found that resting-state prefrontal cortex activity was increased in recent suicide attempters compared to non-attempters (78). Taken together, these findings paint a picture of dysregulated functional connectivity in large-scale brain networks including the default mode, executive control, and salience networks all associated with suicide risk. Of particular note is that several studies implicate dysregulation of salience network structures including the insula and anterior cingulate with suicide risk, lending support to the hypothesis that these structures may be critically important in acute risk for suicide because of their role in switching between the other large-scale functional brain networks (80). Further work is needed to characterize network connectivity in these regions at multiple timepoints in participants at risk for suicide, in order to determine whether these structures are critical for transitioning suicidal thoughts into behaviors.

In addition to measuring large-scale brain network dysregulation during rest, recent neuroimaging studies have also sought to measure neurobiological correlates of SI and suicide attempts in both adolescents and adults using novel tasks that measure social and decision-making processes, given that relational stress and impulsivity have been linked to suicide attempts (50, 83). One study of SI in depressed adolescents categorized ideators into two groups, high- vs. low-SI (based on both recent suicide attempts and SI scores), and used a social interaction paradigm—here, cyberball, a game that varies social inclusion/exclusion—to measure the neurobiological correlates of SI (65). The authors found that adolescents with high SI had significantly reduced activity in the pre- and post-central gyrus, superior temporal gyrus, medial frontal gyrus, insula, and putamen compared to those with low SI, and significantly reduced activity in the caudate and anterior cingulate cortex compared to healthy adolescents (65). Greater SI in adolescents has also been found to be associated with heightened insula activation to social rejection during an EMA protocol (79). In adults diagnosed with mood disorders, suicide attempt was associated with changes in default mode and basal ganglia connectivity during an emotional face-word Stroop Task (67). In addition, during a reinforcement learning paradigm, depressed adults with a history of suicide attempt were found to have reduced ventromedial prefrontal cortex value signaling and disrupted ventromedial prefrontal cortex-frontoparietal connectivity compared to non-psychiatric controls (70).

Drug studies have also assessed neurobiological correlates of rapid-acting anti-suicidal treatment response in adults diagnosed with MDD, though more work is needed in this area to characterize the neurobiology associated with rapid changes in mood following drug administration. For example, changes in SI have been associated with changes in functional connectivity between the anterior cingulate, dorsolateral prefrontal cortex, and superior parietal cortex following administration of low (0.2 mg/kg) and standard (0.5 mg/kg) doses of subanesthetic ketamine (64). In particular, a positive association was found between reductions in SI and increased connectivity between dorsolateral prefrontal cortex and superior parietal cortex at low doses of ketamine, while a negative association was found between reductions in SI and reduced connectivity between left dorsal anterior cingulate and right anterior cingulate regions at standard doses of ketamine (64). The authors posit that these changes in connectivity within anterior cingulate and frontoparietal systems may reflect normalization of function, which potentially contributes to reductions in SI. A second study assessing standard dose ketamine and SI measured salience network connectivity changes between anterior cingulate and insula directly, finding that ketamine administration lowered the membrane capacitance of superficial pyramidal cells (72). The authors suggest that this mechanism could lead to increased bursting of superficial pyramidal cells and increased cortical excitation, perhaps reflecting the mechanism which leads to normalization of connectivity between these salience network regions and other large-scale functional brain networks.

Finally, recent neuroimaging studies with healthy participants have attempted to assess brain regions associated with suicidal thoughts. For example, a recent study that used an adaptation of the suicide-related IAT found increased activity in the bilateral insula, middle occipital cortex, medial prefrontal cortex, and parahippocampal gyri in response to self-death compared to self-life associations (84). A second study measuring connectivity changes between early visual cortex, amygdala, and anterior insula during the suicide-related IAT in a group of healthy participants and small group of recent suicide attempters found that connectivity estimates between these regions correctly categorized recent attempters with 77 to 82% sensitivity and 80 to 85% specificity (85). However, further work is needed to better characterize brain network changes associated with the suicide-related IAT in those with either a recent suicide attempt or current SI, as the previous study included only four participants with a recent suicide attempt. In addition, more research is needed to measure the neural correlates of other suicide-specific behavioral tasks during scanning in order to better characterize the network of brain regions associated with suicide risk.

Taken together, studies exploring the neurobiology of SI and suicidal behavior suggest that local and long-range brain network changes accompany suicide risk. However, significant limitations need to be addressed to better characterize these brain network changes. For example, neuroimaging studies of recent suicide attempters compared with non-attempters will help address acute risk. In addition, measuring neural correlates of cognitive tasks such as the suicide-related IAT will provide important insights into the relationship between suicide risk and network-level changes in brain circuitry, allowing for the characterization of the neurobiology of suicide risk independent of self-report clinical measures. Finally, advances in computational techniques such as the application of machine learning algorithms and mathematical models that characterize decision-making and reinforcement processes will allow for a more complete mechanistic account of the neurobiology of suicide risk.

## Conclusions

Previous research on suicide risk has relied on self-report measures that are not designed to capture rapid, moment-to-moment changes in SI. This impedes efforts to identify phenotypes and biomarkers of the suicidal state and hinders the development of individualized clinical care for those experiencing a current suicide crisis, underscoring the need for improved methodologies capable of capturing rapid changes in suicide risk. Each of the methods reviewed above—EMA, digital phenotyping, cognitive and implicit bias tasks, and candidate neuroimaging biomarkers—can improve our understanding of suicide risk beyond the dichotomy of “suicidal” or “not suicidal.” These methods also offer the potential to reveal whether treatments impact variability in SI, implicit suicide bias, and specific neural circuitry activity as a function of suicide-related stimuli.

While research in this area is ongoing, there are obvious extensions to clinical treatment of patients. Specifically, clinicians have long known that suicide risk exists across a continuum and that active suicide risk ebbs and flows over time. Once these techniques are validated, one can picture how these tools can assist clinicians in monitoring their patients in both rapid-acting interventions or even traditional treatments such as lithium or psychotherapy. For example, digital assessments could provide real-time information about clinical symptomatology in high-risk patients; clinicians could be alerted if their patient has reported increased SI over time, mood fluctuations or reduced sleep as indicative of a “red flag” for active suicide risk. When treating a patient who denies SI, behavioral tasks could provide additional context around implicit suicide risk. Baseline EEG or fMRI scans could provide clues to which treatments might be most effective for a given patient. Therefore, these approaches may define new treatment avenues and lay the foundation for precision psychiatry for the treatment of suicidal individuals.

## Author Contributions

All authors contributed equally to the literature search, writing, and revision of this manuscript. All authors approved the final version of the paper.

## Conflict of Interest

CZ is listed as a co-inventor on a patent for the use of ketamine in major depression and suicidal ideation; as a co-inventor on a patent for the use of (2R,6R)-hydroxynorketamine, (S)-dehydronorketamine, and other stereoisomeric dehydro and hydroxylated metabolites of (R,S)-ketamine metabolites in the treatment of depression and neuropathic pain; and as a co-inventor on a patent application for the use of (2R,6R)-hydroxynorketamine and (2S,6S)-hydroxynorketamine in the treatment of depression, anxiety, anhedonia, suicidal ideation, and post-traumatic stress disorders. He has assigned his patent rights to the U.S. government but will share a percentage of any royalties that may be received by the government. The remaining authors declare that the research was conducted in the absence of any commercial or financial relationships that could be construed as a potential conflict of interest.
